# Longitudinal analysis of factors related to *Helicobacter pylori* infection in Chinese adults

**DOI:** 10.1515/med-2022-0564

**Published:** 2022-11-03

**Authors:** Yan Gong, Yi Luo, Zhilai Chen, Ying Sui, Yansong Zheng

**Affiliations:** Health Management Institute, Second Medical Center & National Clinical Research Center for Geriatric Diseases, Chinese People’s Liberation Army General Hospital, Beijing, China; The 6th Health Department, Second Medical Center & National Clinical Research Center for Geriatric Diseases, Chinese People’s Liberation Army General Hospital, Beijing, China; Health Management Institute, Second Medical Center & National Clinical Research Center for Geriatric Diseases, Chinese People’s Liberation Army General Hospital, 28# Fuxing Road, Haidian District, Beijing 100853, China

**Keywords:** *Helicobacter pylori*, infection rate, follow-up, health management, opportunistic screening

## Abstract

This research aimed to analyze lifestyle-related factors which influence *Helicobacter pylori* (*Hp*) infection and outcomes in Chinese adults. A single-center, retrospective study was performed from January 2012 to December 2020. Self-administered questionnaires were used to collect relevant lifestyle information, and the ^13^C-urea breath test was used to diagnose active *Hp* infection. A total of 18,211 subjects were enrolled in the study, of which 5,511 were females (30.26%). Subjects were studied longitudinally for up to five follow-up visits. At baseline, gastric *Hp* test was negative in 10,670 subjects (58.59%) and positive in 7,541 subjects (41.41%). Males exhibited a significantly higher *Hp* infection rate than females (38.56% vs 2.65%, respectively; *χ* ^2^ = 26.45, *P* < 0.001). Throughout the course of follow-up, *Hp* positive rates in the subjects decreased ( {\chi }_{\text{trend}}^{2}] = 666.04, *P* < 0.001). Among the subjects with baseline negative results, 3–6% changed from negative to positive during follow-up. In contrast, among those with baseline positive results, >70% remained positive, and 21–26% changed from positive to negative. However, only 22–27% of *Hp*-infected subjects received pharmacotherapy. The results indicate the prevalence of *Hp* infection is high in the Chinese population. That additional effort is required to prevent and control *Hp* infection.

## 1 Introduction

*Helicobacter pylori* (*Hp*) is a gram-negative spiral bacillus bacterium that attacks the epithelial lining of the stomach, typically remaining there for many years [1–3]. *Hp* infection is a primary causal factor in numerous gastrointestinal (GI) pathologies including chronic gastritis, peptic ulcer, gastric mucosa-associated lymphoid tissue lymphoma, and gastric cancer [4,5]. In 1994, the World Health Organization/International Agency for Research on Cancer designated *Hp* as a class I carcinogen. Epidemiological reports suggest that the world’s *Hp*-infected population has reached 4.4 billion. Prevalence rates differ significantly by geographical region, with the highest prevalence observed in Africa (70.1%; 95% CI = 62.6–77.7) and lowest in Oceania (24.4%; 95% CI = 18.5–30.4). Among individual countries, *Hp* infection rates vary from 18.9% (95% CI = 13.1–24.7) in Switzerland to 87.7% (95% CI = 83.1–92.2) in Nigeria [6]. Infection rates are significantly higher in developing countries (80%) than in developed countries (30%), and are higher in rural areas than in urban areas. *Hp* infection is significantly affected by lifestyle. One report documented that the *Hp* infection rate in the Chinese population is 40–60%, which places China among the group of highly-infected countries [7]. Indeed, *Hp* is one of China’s most common chronic bacterial infections [8–10]. Among individuals, the conditions which dictate *Hp* infection may change due to treatment or re-infection.

Many unanswered questions remain concerning *Hp* infection, including the likelihood of self-recovery and infection clearance if no treatment is received. Also unknown are the mitigating factors which drive treatment paradigms for infected individuals. There is a critical need for more studies which address these important questions. The ^13^C-urea breath test (UBT) is a gold-standard clinical method used to diagnose an active *Hp* infection [11]. Routine health examinations provide an opportunistic screening tool for identifying *Hp* infection and, to some degree, also can be analyzed to identify disease prevalence in the population [12]. The objective of this study is to investigate the lifestyle factors which influence *Hp* infection rates in the Chinese adult population, as determined by questionnaires and the ^13^C-UBT. This study was performed in a cohort of patients undergoing routine health examinations at a single center over 10 years of follow-up.

## 2 Methods

### 2.1 Study population

All adults (≥18 years) undergoing routine health examination who received a gastric *Hp* test at the Institute of Health Management, Chinese PLA General Hospital from January 2012 to December 2020 were included in the study. A total of 105,623 subject-visits were evaluated, initially. Examinees who received no re-examination or had only one health examination result were excluded from the study, as were those who took oral antibiotics or proton-pump inhibitors within 1 month prior to the gastric *Hp* test and those who underwent a second examination within 6 months. In total, 56,075 subject-visits were excluded, and 49,548 subject-visits were included. Among the included subjects were 18,211 individuals who also were tracked longitudinally for up to five follow-up visits over time. The study protocol was approved by the Chinese People’s Liberation Army General Hospital ethics committee (S2021-636-01). All individuals enrolled were informed that their physical examination data would be de-identified, and signed consent documents.

### 2.2 Clinical evaluations

Basic demographic information including subjects’ age, sex, date of birth, and date of health examination was recorded at first examination, and follow-up duration was calculated based on the length of time between the first examination and subsequent re-examinations. Each follow-up visit recorded whether *Hp* pharmacotherapy was received in the time between the previous and current visit, including standard triple or quadruple pharmacotherapy for *Hp*. A questionnaire was used to determine subjects’ marital status, education level, smoking and drinking habits, and epigastric symptoms including epigastric pain, abdominal distension, hiccup, nausea, and vomiting. If any of these symptoms were frequently present in a subject, he/she was determined to be positive for epigastric discomforts. Marital status was classified to single or married, with the former including never married, divorced, and widowed and the latter explicitly referring to having partners living together. Other important criteria evaluated at initial examination for each subject included the presence or absence of tooth disorders, particularly dental calculus or loose teeth [13]. Halitosis referred to a foul smell in the mouth that was noticeable by the subjects’ themselves, or others.

### 2.3 ^13^C-UBT

^13^C-labeled urea breath test diagnostic reagent (Helikit™, Alta Chem Pharma Ltd, Canada) [4] was used to determine *Hp* infection during examination. Subjects were counseled to not take any antibiotics within 1 month and not eat for at least 4 h before the examination. The diagnostic test required subjects to slowly exhale through a straw into the collection tube for about 4–5 s, upon which the examiner (or subject) tightened the cap immediately. A small volume of drinking water (75 mL) was then added to the plastic bottle containing the ^13^C-urea kit. The solution was mixed until reagent completely dissolved and then drunk slowly by the subjects. Subjects then rested for 30 min with no eating or drinking, and then exhaled again into the collection tube as before. The samples in the two tubes were then analyzed using gas chromatography coupled with the isotope ratio mass spectrometer (Pure Co, Ltd, USA). A change in value of ≥4 over baseline (the initial test) was judged as *Hp* positive, indicating the presence of an active gastric *Hp* infection.

### 2.4 Data processing and analysis

A library was constructed with Epidata 3.0, and data were entered using a double-blinded method. Data were automatically verified and relative errors were checked by trained data entry operators. Statistical analysis was performed using Stata 11.0. Endpoint values were expressed as mean ± standard deviation, and follow-up duration was expressed using median only. Main effect differences in the measurement and numeration data between the groups were calculated by *t*-test and chi-square test, respectively. One-way analysis of variance, chi-square trend test, univariable and multivariable logistics regression analyses all used *P* < 0.05 as the cut-off value for statistical difference.


**Ethical approval and consent to participate:** All procedures in this study complied with the guidelines of the Helsinki Declaration on Human Experimentation. The study protocol was approved by the Ethical Committee of the PLA General Hospital, and written informed consent was obtained from all participants.

## 3 Results

### 3.1 Baseline characteristics of the study population

The clinical and demographic data of the 18,211 subjects at baseline which were relevant for this study are shown in Table 1. Subjects’ age at baseline was 48.06 ± 8.44 years. About 12,700 (69.74%) subjects were male and 5,511 (30.26%) were female. Rates of *Hp* infection were significantly higher in males than in females (44.15% vs 33.10%; *χ* ^2^ = 170.7609*; P* < 0.001). The *Hp*-negative and positive groups were 10,670 (58.59%) and 7,541 (41.41%) subjects, respectively. There were significant differences between these groups in age, sex ratio, education level, smoking and drinking habits, comorbidity with tooth disorders, or halitosis (*P* < 0.001).

**Table 1:** Clinical and demographic data of 18,211 subjects at baseline

Characteristic	*Hp*-negative group (*n* = 10,670)	*Hp*-positive group (*n* = 7,541)	Statistical parameters and *P* value		
			*χ* ^2^		*P*
Age	47.65 ± 8.41	48.58 ± 8.74	7.232	<0.001	
Gender			170.76	<0.001	
Female	3,018 (28.28)	1,493 (19.80)			
Male	7,652 (71.72)	6,048 (81.20)			
Marital status			0.849	0.357	
Single	4,652 (43.60)	3,236 (42.91)			
Married	6,018 (56.40)	4,305 (57.09)			
Education level			868.653	<0.001	
Senior high school or below	3,146 (29.48)	3,846 (51.01)			
Undergraduate	6,253 (58.61)	3,013 (39.95)			
Postgraduate or above	1,271 (11.91)	682 (9.04)			
Smoking habit			33.959	<0.001	
Non-smoker	6,084 (57.02)	4,056 (53.79)			
Ex-smoker	865 (8.11)	542 (7.19)			
Smoker	3,721 (34.87)	2,943 (39.02)			
Drinking habit			68.537	<0.001	
No drinking	4,512 (42.29)	3,051 (40.46)			
Occasional drinking	2,459 (23.05)	1,458 (19.33)			
Frequent drinking	3,699 (34.66)	3,032 (40.21)			
Comorbidity with tooth disorders			751.428	<0.001	
No tooth disorders	6,879 (64.47)	3,342 (44.32)			
Dental calculus	2,713 (25.43)	3,199 (42.42)			
Loose teeth	1,078 (10.10)	1,000 (13.26)			
Epigastric discomforts			345.404	<0.001	
No	8,945 (83.83)	5,465 (72.47)			
Yes	1,725 (16.17)	2,076 (27.53)			
Halitosis			347.907	<0.001	
No	6,977 (65.39)	3,893 (51.62)			
Yes	3,693 (34.61)	3,648 (48.38)			

### 3.2 Risk factor analysis associated with *Hp* infection

Risk factors found to be significantly associated with *Hp* infection status shown in Table 1 were analyzed in a stratified manner and presented in Table 2. Education status is clearly associated with *Hp* infection, as high education level had significantly lower OR of *Hp* infection as compared with low education level. Additional risk factors for *Hp* infection included age, male sex, smoking, frequent drinking, dental calculus, loose teeth, epigastric discomforts, and halitosis.

**Table 2:** Risk factors analysis associated with *Hp* infection in subjects

Characteristic	OR (95% CI)	*Z*-value	*P*-value
Age	1.056(1.043–1.069)	8.64	<0.001
Gender			
Female	Ref		
Male	1.369(1.244–1.533)	6.06	<0.001
Education level			
Senior high school or below	Ref		
Undergraduate	0.675(0.333–1.369)	−1.09	0.277
Postgraduate or above	0.56 1(0.446–0.706)	−4.94	<0.001
Smoking habit			
Non-smoker	Ref		
Ex-smoker	1.336(1.023–1.746)	2.13	0.033
Smoker	1.472(1.363–1.589)	9.89	<0.001
Drinking habit			
No drinking	Ref		
Occasional drinking	1.481(0.730–3.003)	1.09	0.277
Frequent drinking	1.488(1.262–1.756)	4.72	<0.001
Comorbidity with tooth disorders			
No tooth disorders	Ref		
Dental calculus	1.343(1.215–1.484)	5.77	<0.001
Loose teeth	1.316(1.162–1.490)	4.33	<0.001
Epigastric discomforts			
No	Ref		
Yes	1.009(1.000–1.018)	2.05	0.040
Halitosis			
No	Ref		
Yes	1.059(1.021–1.098)	3.11	0.002

### 3.3 Longitudinal assessment of *Hp* infection rates in follow-up

Basic information pertaining to follow-up completion is shown in Table 3. As expected, a small but significant increase in the mean age of subjects during follow-up was observed (*F =* 255.94, *P* < 0.001). Subjects who completed all five follow-up visits were more likely to be male than female ( {\chi }_{\text{trend}}^{2}] = 25.64, *P <* 0.001), and there was a significant decrease in rates of *Hp*-positive tests during the follow-up period ( {\chi }_{\text{trend}}^{2}] = 666.04, *P <* 0.001).

**Table 3:** Follow-up results of *Hp* infection status in the subjects

	Baseline test	The first follow-up	The second follow-up	The third follow-up	The fourth follow-up	The fifth follow-up
Sample size (*n*)	18,211	18,211	7,719	3,587	1,436	384
Age (years)	48.06 ± 8.43	48.06 ± 8.43	51.22 ± 8.19*	52.68 ± 8.22*	54.14 ± 8.28*	55.68 ± 8.66*
Follow-up duration (day)						
Median		485	841	1,150	1488.5	1,893
(P5–P95) interval		279, 1,480	572, 1,819	824, 2,330	1,056, 2,301	1,063, 2,568
Min. and Max.		181, 42,455	370, 42,702	545, 42,667	736, 43,006	920, 2,779
Gender						
Male (%)	12,700 (69.74)	12,700 (69.74)	5,523 (71.55)*	2,598 (72.43)*	1,039 (72.35)*	301 (78.39)*
Female (%)	5,511 (30.26)	5,511 (30.26)	2,196 (28.45)	989 (27.57)	397 (27.65)	83 (21.61)
Hp infection						
Negative (%)	10,670 (58.59)	12,039 (66.11)	5,478 (70.97)	2,678 (74.66)	1,106 (77.02)	289 (75.26)
Positive (%)	7,541 (41.41)	6,172 (33.89)*	2,241 (29.03)*	909 (25.34)*	330 (22.98)*	95 (24.74)*

^*^When compared with the baseline or the first follow-up results, *P* < 0.05.

### 3.4 Changes in *Hp* infection status during follow-up

A summary of the overall changes in *Hp* infection status of the 18,211 subjects is shown in Table 4. The data suggest that of those subjects who tested negative at baseline, 94–98% remained negative after a median of 1,893 days of follow-up, and 3–6% went on to test positive for *Hp* infection. Conversely, of those who tested positive at baseline, 74–79% remained positive and 21–26% went on to test negative during follow-up. Interestingly, the total percentage of subjects changing from *Hp*-positive to negative during follow-up was significantly greater than that changing from negative to positive (*n* = 890, 20.92% vs *n* = 2,822, 10.56%; *χ* ^2^ *=* 1,500, *P <* 0.001). This was the case for each follow-up visit, where the percentage of subjects changing from *Hp*-positive to negative was significantly higher than that changing from negative to positive (*P <* 0.001).

**Table 4:** Changes in *Hp* infection status at each follow-up visit from baseline

Finished follow-up visit	Number of subjects	*Hp*-negative at baseline (10,670 subjects)		*Hp*-positive at baseline (7,541 subjects)		Statistical parameters and *P* value	
		Keeping negative (%)	Changing to positive (%)	Keeping positive (%)	Changing to negative (%)	*χ* ^2^	*P*
First	7,541	10,121 (94.85)	549 (5.15)	5,623 (74.57)	1,918 (25.43)	1,600	<0.001*
Second	2,583	4,921 (95.81)	215 (4.19)	2,026 (78.44)	557 (21.56)	575.20	<0.001*
Third	1,063	2,443 (96.79)	81 (3.21)	828 (77.89)	235 (22.11)	332.52	<0.001*
Fourth	377	1,021 (96.41)	38 (3.59)	292 (77.45)	85 (22.55)	127.59	<0.001*
Fifth	115	262 (97.40)	7 (2.60)	88 (76.52)	27 (23.48)	43.50	<0.001*

^*^Comparison of percentage of subjects changing from negative to positive versus that from positive to negative at each follow-up visit, *P* < 0.05.

### 3.5 Comparison of treatment

Of the 7,541 subjects who were *Hp*-positive at baseline, the infection status outcomes in subjects who received standard triple or quadruple therapy are shown in Table 5. At each follow-up visit, the percentage of *Hp*-infected subjects receiving pharmacotherapy was 22–27%, and those not receiving pharmacotherapy was 73–78% (*P <* 0.001). Of the subjects receiving pharmacotherapy, the percentage of subjects changing from *Hp*-positive to negative remained 93–96% throughout follow-up (*χ* ^2^ *=* 1.09, *P =* 0.895). Of those who did not receive pharmacotherapy, the percentage of subjects changing from positive to negative was 0–0.05%.

**Table 5:** Effects of pharmacotherapy on subjects who were *Hp*-positive

Follow-up visit	Subjects (*n*)	Receiving no pharmacotherapy			Receiving pharmacotherapy		
		Keeping positive (%)	Changing to negative (%)	Total (%)	Keeping positive (%)	Changing to negative (%)	Total (%)
First	7,541	5,532 (99.95)	3 (0.05)	5,535 (73.40)	91 (4.54)	1,915 (95.46)	2,006 (26.60)*
Second	2,583	1,997 (99.95)	1 (0.05)	1,998 (77.35)	29 (4.96)	556 (95.04)	585 (22.65)*
Third	1,063	814 (100.00)	0	814 (76.58)	14 (5.62)	235 (94.38)	249 (23.42)*
Fourth	377	287 (100.00)	0	287 (76.13)	5 (5.56)	85 (94.44)	90 (23.87)*
Fifth	115	86 (100.00)	0	86 (74.78)	2 (6.90)	27 (93.10)	29 (25.22)*

^*^ *P <* 0.001 for the percentage of subjects receiving treatment versus that receiving no treatment.

### 3.6 Analysis of influencing factors of receiving treatment

Of the 7,541 subjects who were positive at baseline, 2,006 subjects received pharmacotherapy and 5,535 received no pharmacotherapy between the initial examination and first follow-up. Factors influencing the subjects’ decision to receive pharmacotherapy were epigastric discomforts (OR = 1.488, *P <* 0.001, 95% CI: 1.262–1.756) and halitosis (OR = 1.343, *P <* 0.001, 95% CI: 1.215–1.485), as determined by the stepwise multivariable logistics regression analysis of the questionnaire.

## 4 Discussion

A number of studies have been performed on *Hp* infection rates in China, the consensus of which have reported a *Hp* prevalence rate of 40–60% [7], meaning that almost one out of every two individuals is infected. However, most of these studies are cross-sectional in nature, and cohort studies have been rare [14–18]. Prior research has confirmed that chronic gastritis, peptic ulcer [19], and gastric cancer [20] are closely associated with *Hp* infection, and ∼1% of infected individuals eventually progress to have gastric cancer [21,22]. In contrast with the numerous probiotic microbiota that colonize the GI system, *Hp* are pathogenic microbes, and *Hp* eradication is an important step in preventing gastric cancer. Eradication at the superficial gastritis or asymptomatic stage has been shown to have the most significant benefit. Health benefits of *Hp* eradication outweigh the risks, as there is no evidence of an association between *Hp* eradication and disease risk [23]. The optimal time for *Hp* eradication appears to be prior to the onset of gastric mucosal atrophy and intestinal metaplasia [24]. Professor Correa, an American pathologist, believes that *Hp* eradication prior to onset of gastric mucosal atrophy and intestinal metaplasia can prevent the development of intestinal gastric cancer by nearly 100% [25]. Even after the onset of intestinal metaplasia, *Hp* eradication still contributes to the repair of gastric mucosa by helping to maintain the intestinal status quo and prevent progression to gastric cancer [26].

One thing which remains clear is that *Hp* pathogenicity in humans is influenced by both the duration of infection combined with individual susceptibility. Factors associated with high *Hp* prevalence rate observed in many countries worldwide include hygiene, dietary hygiene, living habits, and even housing conditions.

This study found that the *Hp* infection rate was 41.41% in Chinese adult subjects at our institution and was significantly higher in males than that in females. We used the non-invasive ^13^C-UBT which remains the gold-standard test to confirm *Hp* infection, having sensitivity and specificity greater than 95% [11,27]. The population of this study is unique, having a higher overall income and health awareness, which probably explains why the *Hp* infection rate was on the lowest end of those previously reported. However, the infection rate in this cohort was higher in males than in females, consistent with previous studies [28]. A large-scale prospective case–cohort study in the Chinese population recently reported that the *Hp* infection rate was significantly higher in urban populations, or those with higher education levels, but no significant differences were observed between sexes or across the age spectrum [29]. In contrast, in the present study, education level was determined to be associated with a lower *Hp* infection rate, and age, male sex, smoking, frequent drinking, dental calculus, loose teeth, epigastric discomforts, and halitosis were additional risk factors in *Hp* infection.

Our previous study reported that males are more susceptible to *Hp* infection, and that efforts to maintain proper oral hygiene and prevent oral disorders, particularly dental calculus and loose teeth, are essential for avoiding or eradicating *Hp* [13]. Moreover, we and others have consistently shown that *Hp* prevalence increases with age [30,31]. One surprising outcome of the present study is that, despite the subjects’ age and male percentage increasing over time during follow-up, the *Hp* infection rate decreased ( {\chi }_{\text{trend}}^{2}] = 666.04, *P <* 0.001). This contrasts with what is expected based on previous reports. Additionally, the percentage of subjects changing from *Hp*-positive to negative during follow-up was significantly higher than that from negative to positive (*χ* ^2^ = 1,500, *P <* 0.001). We believe that a principal reason for this could be due to the increase in individual awareness of the dangers of *Hp* infection, thus leading to more individuals seeking pharmacotherapy. From a healthcare perspective this could be viewed as a positive development because it indicates that *Hp* testing during routine health examination is having a beneficial impact on public health.

At each follow-up visit, the percentage of subjects receiving no pharmacotherapy (73–78%) remained significantly greater than that of subjects receiving treatment (22–27%, *P <* 0.001). Among the untreated subjects, vanishingly few (0–0.05%) reverted from *Hp*-positive to negative, and the vast majority remained positive. Also of interest was the percentage of *Hp*-negative subjects (∼3–6%) who subsequently tested positive upon follow-up after 485 days (1.33 years) or 1,893 days (5.19 years). This suggests that *Hp*-negative people may become infected, or previously treated patients may become re-infected if they do not take precautions. Clearly there is much to be learned regarding mechanisms of *Hp* infection and the means to prevent its occurrence.

The results of the present study suggest that pharmacotherapy is highly effective in *Hp*-positive patients, with an eradication rate >90%. Indeed, pharmacotherapy may be the principal reason for the decline in *Hp* infection rate observed in the subjects over time. Throughout the course of follow-up, 70% of individuals who were *Hp*-positive at baseline remained positive, even up to 2,779 days of follow-up. At the same time, a subset of *Hp*-negative people has the potential to become infected. Based on this evidence we conclude that health examination plays an important role in the screening, prevention, and control of *Hp* infection, especially for *Hp*-negative adults. Furthermore, it is of great practical importance to take active measures to prevent and control *Hp* infection. For the *Hp*-positive population, the presence of frequent epigastric discomforts (OR = 1.488, *P <* 0.001, 95% CI: 1.262–1.756) and halitosis (OR = 1.343, *P <* 0.001, 95% CI: 1.215–1.485) were significant factors which influenced their decision to seek pharmacotherapy. The low percentage of subjects receiving pharmacotherapy suggests that efforts to re-enforce health education are vital for encouraging asymptomatic populations to seek therapy.

Our study was strengthened by the large size of the cohort examined (18,211), combined with the duration of follow-up in this cohort. Intervals of at least 6 months between each follow-up visit prevented the potential for error caused by re-examination within a short period after pharmacotherapy. *Hp* test results were dynamically monitored over time in each subject that received a follow-up examination, which allowed us to discern the duration and outcomes of *Hp* infection in this population. These results will inform healthcare practitioners and provide guidance for screening and managing *Hp* infection in the population.

A notable limitation to this study is that a substantial number of subjects were lost to follow-up after the second visit, such that only ∼2.11% of the original 18,211 completed all five follow-up visits, because physical examinations concerned were opportunistic and optional, the loss of follow-up was more severe in this retrospective study. Given this limitation, this study can only confirm that >70% of the *Hp*-positive population remained positive in a median follow-up duration of 1,893 days. While this finding does not necessarily suggest that *Hp* infection will last forever in the absence of therapy, it does suggest that the possibility of self-recovery after *Hp* infection is relatively low. As such, individuals should be counseled on the long-term benefits and advantages of *Hp* eradication.

In conclusion, we demonstrated that the prevalence of *Hp* infection is high in the Chinese adult population. Aggressive pharmacotherapy consistently eradicates *Hp* at a high clearance rate, but this outcome is limited by the fact that <30% of all *Hp*-positive patients receive therapy. Rates of *Hp*-positivity remain high in the absence of pharmacotherapy. Thus, it is clear from this study that additional effort is required on the part of healthcare providers and their patients, to prevent and control *Hp* infection in the long term.
